# Recovery in Two Cases of Acute Traumatic Tetanus

**Published:** 1864-12

**Authors:** O. Foster

**Affiliations:** Hitchin


RECOVERY IN

### TWO CASES OF ACUTE TRAUMATIC TETANUS.

By O. FOSTER, Esq., M. R. C. S.

In looking over my case-book I am reminded of the great interest I took in two severe cases of acute traumatic tetanus under my care within the last two or three years. I do not lay claim to any originality in the treatment of them, but as it was successful I forward them for insertion in the *Lancet* If perchance by my remarks any one should be happier in the saving of life or mitigation of suffering in that formidable malady I shall be compensated fiftyfold.

It having fallen to my lot during a career of thirty years' country practice and surgeoncy to a small provincial hospital to meet with several cases of acute traumatic tetanus which were unsuccessfully treated with morphine, belladonna, aconite, tobacco, warm baths, counter irritation, &c., singly and combined, and in some of which, I fear, the remedies increased the sufferings rather than relieved the sufferer, if they did not even hasten a fatal termination, I determined, if ever another case fell under my charge, I would act more upon the expectant principle than I had hitherto done, attending only to the usual routine of keeping the bowels well open, at the same time supporting the patient to the best of my power, and leaving the prominent features of the malady almost entirely uncared for. Singularly enough, it was not very long before the two following severe cases occurred in our little hospital of thirty beds. Both of them being my patients, I had the opportunity of carrying out my intention, and was rewarded by coaching them both through a severe and dangerous illness to a state of convalescence and health.

On the 9th of May, 1861, John S——, aged twelve years, a farm laborer, received a contusion of the shoulder in consequence of being jammed against a gate-post. On the 15th he was sent to the hospital in a tetanic condition; there was no external wound or evidence of injury to the bone. Trismus was severe, and the least effort to open his mouth was accompanied by violent spasms in the neck, which soon extended to the back and legs; indeed the whole of his muscular system became so affected that it was at times difficult to keep him on the bed. Such were his sufferings that his near relatives only desired the one speedy termination to his misery. This state of things lasted for nearly three weeks, at the expiration of which time the severity of the symptoms began to subside, and he gradually advanced towards convalescence, which was slow. He left the hospital on July 3rd quite well. The treatment consisted of half a grain of acetate of morphia every night; castor oil and spirits of turpentine, half an ounce of each every morning, which acted freely, bringing away a quantity of vitiated offensive matter. The effort to swallow produced such violent spasms that we were often unable to get down the amount of nourishment required; but the directions were to give as much strong beef-tea, milk and port-wine as he could take, and we sometimes succeeded in getting down nearly half a pint of each in the twenty-four hours, of course taken in very small quantities.

Noah C———, aged forty-five, in good health, and a prudently living man, was admitted on the 21st of March, 1862, having that day had his wrist and hand crushed when attending to some machinery. I removed the arm below the elbow, and the case progressed most favorably for ten days. On the 31st he had a rigor, which was followed by stiffness in the jaw and neck, and was attributed to cold. On April 1st, I strongly suspected what was impending, and on the 2nd we had well-marked evidence of tetanus. The case was a severe one, and for three weeks he suffered as much as I had ever witnessed on like occasions. The spasms were excessively severe. Opisthotonos existed to such an extent that he often only touched the bed with his head and heels. From the inordinate action of the masseter and temporal muscles, as well as those of deglutition, it was impossible to administer nourishment—which consisted of strong beef tea, milk and port wine—except in small quantities; of the latter he sometimes took a pint in the twenty-four hours. The general opinion was that he must die, though I was encouraged by the successful issue of the former case to hope otherwise; nor was I disappointed. On or about the 20th of April the case showed signs of amendment, which gradually though slowly progressed towards convalescence, and he left the hospital well on the 20th of May. The treatment carried out was the same as in the case of the boy S——, with the addition of the inhalation of chloroform, which afforded relief only during the first two days; and that of a blister to the spine, which decidedly increased the severity of the spasms and his misery. The only medicine given was a grain of acetate of morphia every night, and half an ounce each of castor oil and spirit of turpentine every morning, which was steadily persevered with, and brought away, as in the former case, a quantity of vitiated secretions.

It will be evident to the reader that the tendency of the report of these cases, and my object in giving publicity to them, is to condemn the administration of powerful medicines, or resorting to active measures in the treatment of acute tetanus. We have here evidence in two instances of the subsidence of severe morbid irritation, the physical powers not having been crippled by depressing remedies.

Hitchin, Sept. 1864.

---

—Earthquakes on the Pacific coast are likely to take a place in E iology. We have known a number of instances, in the present year, in which the effect of the shock on the nervous system of sensitive females has been distressing, if not serious. In one case labor was prematurely induced, and in a second the same result was threatened. There is no such thing as getting inured to them. On the contrary, the dread of them rather increases. The Mexicans and South Americans, who have had sad experience of earthquakes in their native country, suffer the most from them here. There appears to be no serious ground for apprehension, however. In a few localities, especially in the southern counties of California, there have been undulations of the earth sufficient to endanger or destroy buildings of a certain class. But we have no authentic account of earthquakes capable of doing serious mischief having occurred in the locality of San Francisco, or anywhere to the northward.—*San Francisco Medical Press.*

